# New York Odontological Society

**Published:** 1894-10

**Authors:** 

**Affiliations:** New York Odontological Society


### Reports of Society Meetings.

### NEW YORK ODONTOLOGICAL SOCIETY.

A REGULAR meeting of the New York Odontological Society was held on Tuesday evening, June 19, 1894, at the New York Academy of Medicine, the President, Dr. Brockway, occupying the chair.

The minutes of the previous meeting were read and approved.

### INCIDENTS OF OFFICE PRACTICE AND CASUAL COMMUNICATIONS.

*Dr. Bogue* (having exhibited three teeth slightly eroded).—These results of an experiment, which I have shown you before the meeting, is a repetition of one which I saw performed by Dr. Michaels, of Paris. He made the extraordinary assertion that sulphocyanide of potash would produce the erosion which we frequently see upon teeth, and for which we have been unable, ordinarily speaking, to find a cause or a cure. He showed me two capillary siphons at work, one drawing water from a bottle onto a tooth, and the other taking the water back to another bottle. The other day, after the little discussion which we had at our last meeting, I took sulphocyanide of potash, which, by the way, I got at Eimer & Amend's, and, dissolving one part in a thousand of water, let it drop upon these three teeth by means of the siphon, drop by drop, so that from Saturday morning until a few minutes ago there has been a constant dropping upon these teeth. You will have noticed that the erosion is plainly distinguishable on these teeth. If any gentleman cares to repeat these experiments, I will take the liberty of asking him to look with the microscope very carefully at some specimens of natural erosion, and then compare them with teeth acted on by the brush. He will perhaps see the same polished surface as is exhibited in one of the teeth here. Sulphocyanide of potash gave a slightly acid reaction to my litmus paper, after it had been lying wet all night. It is found always in the saliva, and nowhere else in the body. I have seen three analyses, one of them giving six-tenths of one part in a thousand, another seven-tenths, and another nine-tenths. I am very glad to bring this experiment before you, giving the credit of it to Dr. Michaels, and hope some other gentleman may see fit to take up this matter and continue the experiments and study.

*Dr. Starr.*—May I ask how you use the siphon, whether you let it drip directly on the teeth?

*Dr. Bogue.*—Yes. The end of the tube was not much larger than the end of your pencil. It dropped the fluid on the teeth continually. Dr. Michaels's apparatus was different. He took only one tooth, and as the drop fell onto the tooth, another siphon took it away and carried it into another bottle. This experiment only lasted from Saturday morning until before I came to the meeting to-night. I believe the teeth are still wet as when I took them out. I looked upon it last summer as a discovery of great importance. I tried to have it brought before this Society, but was unsuccessful. The secretary's remarks upon the erosion proceeding in the manner in which it has showed that it could not be due to wear.

*The President.*—I have long been satisfied that the tooth-brush theory did not account for the erosion we have seen.

*Dr. Howe.*—That experiment is exceedingly interesting and very valuable, and suggests the agent by which possibly the solution is effected in erosion. I want to contribute a little fact on the subject that came under my observation within a week or two. I saw erosion for the first time, I think, on the deciduous teeth of a child six years and four months of age, affecting the cusps of the cuspids and premolars. The child had always been, and was then, very healthy. Both parents of the child have erosion on their teeth, the father's being extensively wasted.

*Dr. Francis.*—Did the surfaces present a polished appearance?

*Dr. Howe.*—Yes. They presented a smooth polished appearance, in cup-shaped depressions, and the teeth were without decay.

*The President.*—I have seen such cases in temporary teeth.

*Dr. Bogue.*—One of the gentlemen asked, a moment ago, if sulphocyanide of potash produces this effect on one tooth, why it does not produce it on all the teeth in the mouth? I think the question would naturally be borne in mind by all investigators; and with it would come the question as to whether the presence of a greater or less amount of the substance in the saliva has an appreciable effect on the health of the patient. I think in all cases of erosion we find a lower condition of vitality than what we would regard as normal. I do not know that I am correct, but I venture to call attention to the points that happen to occur to me. It may be true that we have in this affection a diagnostic symptom of importance.

*Dr. Howe.*—I would like to ask Dr. Bogue if he meant to raise the question whether a variable amount of this compound in the mouth had an effect on the health of the patient, or whether it was that a greater or lesser amount of the sulphocyanide of potash in the saliva was an evidence of health being better or worse.

*Dr. Bogue.*—The latter is what I meant. It is a diagnostic indication.

*Dr. Howe.*—I understood him to raise the question whether this agent had an effect on the health.

*Dr. Bogue.*—I should not dare to put it that way just yet. We do not know enough about it.

Dr. Smith then read a paper on a "Method of manipulating Gold, as practised Fifty Years Ago."

(For Dr. Smith's paper, see page 627.)

Dr. S. B. Palmer, of Syracuse, also read a paper, entitled "Review and Revision of Practice."

(For Dr. Palmer's paper, see page 610.)

DISCUSSION.

*Dr. Palmer.*—I would say that much of my paper cannot be understood by a hasty reading, nor could I put twenty-five years' practice into so short an article to have it understood. So far as this subject is concerned, I shall not be annoyed at any question that may be asked.

*Dr. Littig.*—You spoke of lining the cavity with tin before putting in the amalgam. Would an amalgam of pure tin answer the same purpose?

*Dr. Palmer.*—It would be a good amalgam, but it is so soft it would wash out. Still it would be an improvement as a conductor. If you know the law, silver stands at a conductivity of one thousand and tin at one hundred and forty-five, so there is a vast difference.

*Dr. C. E. Francis.*—Dr. Palmer has given us a very valuable paper, which bears evidence of much careful study, observation, and experiment. I, for one, feel thankful to him for his excellent suggestions, many of which I think I can carry into practice.

Dr. Palmer's reference to the use of amalgam in poorly calcified teeth reminded me of a case which came into my hands to-day. A young gentleman called on me to have his teeth attended to. His sixth-year molars had been filled on their grinding surfaces with amalgam. The teeth were of a low-toned character and the fillings proved a failure. I could pass an excavator between the fillings and cavity margins, where I found the dentine much softened. On the buccal surfaces of the same molars were very small fillings of copper amalgam, which were in better condition and preserving the cavities.

I have but little confidence in the employment of amalgam in children's teeth, either temporary or permanent; for observation has convinced me that three-quarters of them give out. Neither do I recommend gold for poorly calcified teeth, especially in the mouths of children, much preferring gutta-percha, oxyphosphate of zinc, or tin-foil.

Many years ago a young daughter of one of my old patients happened to fall into the hands of Dr. F. B. Smith, who is present to-night. Her teeth were much impoverished and many of them decaying. Dr. Smith filled them with gutta-percha, and they have since been refilled with gold. She is now a middle-aged lady, but still retains her teeth, and I think their salvation was in great part due to the gutta-percha stoppings introduced by Dr. Smith just at the right time.

Permit me to mention another instance showing the superiority of gutta-percha over gold in young teeth. Some years ago a young school-girl called upon Dr. Carr (who was associated with me at that time) to receive attention. One of her superior lateral incisors had been filled some two or three years previously on both posterior and anterior approximal surfaces. One filling was of gutta-percha and the other, a very tiny one, of gold, both inserted at the same time by Dr. E. J. Dunning, who was well known as an excellent operator and possessing good judgment. He evidently considered the walls of the small cavity sufficiently dense to justify the use of gold, but it failed. The tooth-structure softened around it and the cavity was much increased in size. The gutta-percha filling, however, had perfectly preserved the other cavity, which required no further excavation. You will observe that here were two cavities in the same tooth and filled at the same time by the same operator.

*Dr. Watkins.*—How old was the child?

*Dr. Francis.*—About ten or twelve years of age, as near as I can recollect.

*Dr. Watkins.*—I have enjoyed Dr. Palmer's paper very much indeed. It is very instructive, and I feel that I have learned many things from it. However, there are one or two things that I want to say. One is in regard to using tin-foil under amalgam or gold fillings. It answers a twofold purpose. One thing which the doctor did not mention was that it is a poor conductor, and where the tooth is very sensitive the changes of temperature will affect it very much less than if it were filled entirely with gold or amalgam. I have frequently practised that in large deep cavities, where there was excessive sensitiveness, with very beneficial results. I was very glad to hear the doctor put emphasis on a remark which I made before this or the First District Dental Society, a few months ago, in regard to putting in combination fillings of tin and gold, insisting that where tin and gold is used, the major portion of the filling should be of tin, otherwise tin will be destroyed if it be the smaller portion. In regard to filling with amalgam, as long as we have been using amalgam it seems to me that we do not use it as intelligently as we should. I believe it is not taught in our colleges as it should be, and in discussions in societies it is much neglected. We go away with impressions which are entirely misleading. I believe that in putting in an amalgam filling, it is just as necessary to make that a perfect filling as it is in putting in gold. It is just as necessary to take pains with it in every particular, just as necessary to keep it dry, just as necessary for us to use our very best efforts, and I believe that is seldom done. Consequently we have failure with amalgam fillings. I believe as much as I believe anything that when amalgam is placed in the cavity that the rubber dam should be applied, because I think any man who can put in a filling at all, can do better work with the dam than he can without it. Where the rubber dam is applied, you have sufficient time to pack the amalgam properly and work the mercury out of it, and the cavities should not only be filled to the outer surface and finished off, but should be filled again and packed again and again, at different times, and the mercury drawn to the top, so you can cut down all the surface, whether it be a compound cavity or a simple cavity, until you come to good solid amalgam, and have the amalgam as near the same consistency entirely through the filling as it is possible. And if you do that there will be very little change. Therein lies the secret of success in filling with amalgam. You will have clean, dry fillings without oxidation, and they will stay and last to a remarkable extent. If fillings were put in that way, you would hear less about failures. As a rule, they are put in very differently, I think.

The doctor spoke of filling canals with gold or platinum wires.

That method has been used by Dr. Morrison, of St. Louis, for many years. He carries chloro-percha into the canal, and then forces the piece of gold wire, which has been previously fitted to the root, into the apex, twists it off, and allows it to remain there in that way, hermetically sealing the canal, and he is sure that he has something there which will not change in the cavity.

*Dr. Howe.*—This paper of Dr. Palmer's is a very important contribution to our literature, in that it presents a theory of the etiology of caries. This subject is often regarded as settled. Dr. Palmer suggests thoughts that seem to unsettle it. When Dr. Palmer says that oxygen is the active agent in producing caries, I suppose he means that oxygen is the agent that produces the acid. I do not feel prepared to discuss this subject, but some thoughts and facts present themselves to my mind that without preparation I will try to express as best I can. Dr. Palmer has referred to antiseptics as a remedy for caries, and I would like to raise the question, How much have we found antiseptics to be a remedy? The acme of antiseptic influence, for the prevention of recurrence of caries is presented, I suppose, in a copper amalgam filling, since Dr. Miller has shown that it exerts a continual antiseptic influence. Micro-organisms cannot proliferate nor exist in contact with it, nor for a certain distance around it. I suppose we all have observed, however, that decay recurs at the margin of these fillings, and proceeds to disintegrate the tooth right in contact with this antiseptic material. On the other hand, tin-foil, gutta-percha, and amalgam are—on occasion—placed into cavities which are poorly prepared, from which decayed bone is sometimes not all removed, and the material perhaps placed in wet,—and yet teeth thus filled are preserved from recurrence of decay, sometimes for long periods of time. The former fact seems to show that antisepsis is not efficient as a prophylactic, and the latter facts indicate to my mind that other influences than antiseptic ones may be sufficient to arrest and inhibit decay. I would like to ask Dr. Palmer to tell us why a tin-foil or gutta-percha filling, or even an amalgam filling, put in under such difficulties as result in leaving decay in a cavity, and introducing the material in a wet condition, is so often efficacious for a considerable time. We do know that such things occur. What do these facts mean in regard to the theory that Dr. Palmer has suggested?

*Dr. Palmer.*—Those are very fine points, but the theory perfectly explains them. The first point that Dr. Howe mentioned was about copper amalgam, which we all know preserves teeth very well under certain conditions,—that is, when the two elements unite harmoniously. Where copper amalgam comes against enamel, the enamel will be wasted away, because of the principle upon which it acts,—the galvanic action around the copper amalgam with the saliva between it. Why does it not dissolve the dentine? Simply because the oxide has been absorbed into the dentine, taking the place of the lime that has been extracted, and has thus created an equal in potential. If you can produce an oxide of the filling, and have it absorbed into the dentine, there is no action between the two. The potentials are one. As soon as the amalgam filling is introduced, if the tooth is very porous, an action is set up in the amalgam,—that is, on the surface against the tooth the lime is soon dissolved out. You have then the same trouble as you would have with a gold filling. The dentine has not received enough oxide to satisfy and fill up the pores, and the dentine is a positive element. Dr. Brockway spoke of the deciduous teeth, which are very frail, and it is almost impossible to use the amalgam in those teeth. If you do use it, it would be best to have a lining of something between it and the tooth. Amalgam shrinks by the galvanic action upon the surface of the plug next to the dentine. The only amalgam fillings that I removed that were bright were those in which the teeth were lined by a heavy varnish. In my paper I do not go into general practice any more than I can help.

One of the gentlemen spoke of the union of tin with gold. I have practised that until I know how far mutual induction will take care of tin with gold. Gold will penetrate the tin to the thickness of one layer to two of tin-foil. That will preserve it forever. It is an alloy, not an amalgam, but the principle would be the same. If you line a cavity with three or four thicknesses as a guard filling, that portion that is preserved by the gold entering into it will be about one-half of it, and the other will turn black from the decomposition of the dentine back of it. There will be one or two layers of foil that will turn black and be soft. If you put in one-quarter or three-quarters of the tin, then there is no disolving of the tin whatever. There is no extra dissolution of the tin, any more for having the gold with it than there is in having gold with the amalgam. It causes a rapid oxidation which ceases as quick as the oxide enters the dentine. It is a poor conductor, and we gain much in that. We gain another thing. It is easier moulded. We gain chemically by the oxidation of the tin, and it makes a preserving filling. Those are scientific points, and

I think they will answer very well what you have brought out. I took a gold plate and placed upon it some tin-foil, put it in a vulcanizer and vulcanized it. When I took it out the tin appeared black. I brushed it off, and it was bright tin. It was perfectly affixed to the gold. It contained gold, or the gold imparted to it a preserving influence that we call induction. It would not be corroded by any ordinary action in the mouth. When tin and gold are in contact the tin will be slightly oxidized. We want to know what we are doing. Why I adhere so closely to chemistry or to the science of it is that if we learn to do a thing twice, we can do it as often as we wish.

I wish to stand free from any charges against any other theory, as I contend that there are no two theories upon this subject. The fermentation is a fact and a condition. We are getting at it. Where fermentation comes in, it produces an acid and decay. The idea I present is that we can fill the dentine with an oxide. Oxide is recognized every time where there is any oxidation going on. If it is sealed up in the dentine, there is no more oxidation and no more decay. I have nothing to gain or claim through this, except for the science of dentistry. I want to know for myself, and I want the profession to know, whether I have been writing all these twenty-five or thirty years upon a subject that does not amount to enough to be mentioned.

*Dr. Watkins.*—I would like to ask Dr. Palmer if he has not noticed in those tin and gold fillings, where the majority of the filling is made of tin, that instead of the tin deteriorating as it does where there is a small quantity of the tin in the cavity and exposed, that the filling will take on itself more the form of an amalgam and become hard instead of being soft.

*Dr. Palmer.*—The surface of that filling has undergone a chemical change, and has formed an oxide that is much harder than tin. If you will go through it, you will find the same tin there. In regard to mixing tin and gold together, I can do that successfully. I got that idea from Dr. Abbott, in Berlin. Some fillings I have seen for years, in the mouth of the sister of Dr. Nash, look like amalgam, but they are harder, and there is now and then a pit in it, and sometimes a streak of gold. I experimented to find out why those pits should be in the tin. Mix the tin and gold in layers. If you fold two or three layers and it comes to the surface, there will be a pit. If you make a stack of layers of tin and gold, and use it in ribbons or cylinders, then each gold leaf will preserve one of tin. If you put it in wet, you can do so. If you place it in without being wet, it has to undergo the electrical change, as the filling wears down; but in either case you have an absolute mixture. If you add more tin, you will have it dissolved out by electrical action. I have practised this so much that I know these to be facts.

*Dr. Howe.*—Will Dr. Palmer tell us what is the potential relation of gutta-percha to dentine?

*Dr. Palmer.*—I should think it was about equal. Conductivity would be a little more with so much of the oxide in it. Dentine is only 15. It would stand up to about 20 or 25. It depends on the amount of conducting matter that would be in the filling. There is more in the preserving properties of gutta-percha than I once supposed, so far as allowing nature to recalcify is concerned. Nature works from within upon the alkaline or positive pole of a battery. It works from the inside out, and nothing interrupts that. An acid is an outside influence. Dentine is formed in the pulp-chamber. Why is that so? Nature sets to work from the pole in which it works on the alkaline side, but if you set up any irritation there with an acid from without, you turn the current back. If you turn that back, there is no repair from within.

*Dr. Howe.*—Does Dr. Palmer mean to say there is no repair on the part of the pulp in response to the irritations of the tin?

*Dr. Palmer.*—What I mean to say is that an acid irritation, the same as produced by fermentation in the cavity, turns the current back so far as nature is concerned.

*Dr. Howe.*—I supposed that secondary dentine was deposited by the pulp in response to the irritation.

*Dr. Palmer.*—We find it to be a fact that the pulp-chamber is filled with secondary dentine caused by irritation, and the cavity can go on into the pulp-chamber; but it requires a long period to do it.

*Dr. Howe.*—The process of decay generally overtakes the pulp. Secondary dentine is not built up fast enough.

*Dr. Palmer.*—That is a point of whether the acid turns back the current instead of allowing it to continue. If you take out the metal fillings, and put in gutta-percha, that will allow nature to work. You take a gold filling and place it close to the pulp; it may last fifteen or twenty years, but as soon as an irritation takes place, and the filling is removed, there is nothing but the organic matter over the pulp. It has taken away from it what there was at first. There is something of the reversal of the current that is detrimental. Most operators are reminded of cases which seemed perfect, where there was an irritation that produced an acid that reacted.

*Dr. Bogue.*—Where you find the clasps wearing through, there you find the secondary dentine.

*Dr. Palmer.*—You do not find it so much in cavities.

*Dr. Bogue.*—Thinking of what you said, that illustration comes to my mind.

*Dr. Palmer.*—Anything of a wearing nature that is not too rapid will cause that. That is done by another action,—not so much by reaction as by attrition.

*Dr. Bogue.*—I understood you to say that oxygen was the element in all cases, and is at the bottom of dental decay. I also understood you that oxygen is the element which is at the bottom of a process that takes place in compound fillings and prevents decay. Am I right?

*Dr. Palmer.*—Oxygen destroys dentine, which is a conductor or positive element; an oxide renders dentine negative. You are correct in the point that oxygen is the thing to prevent decay, because it oxidizes that part which the acid would attack. You did not fully understand the meaning of the term. Wherever the potentials are equal, there is no acid formed, and consequently no decay.

*Dr. Bogue.*—The word potential means latent power. Applying it to galvanic action, will you explain it?

*Dr. Palmer.*—Wherever there is a piece of metal placed in, the mouth immediately assumes what I call a potential. By attaching the galvanometer needle to it, in connection with the mucous membrane, it turns the needle suddenly. In a short time it runs down. That is a law that occurs with all metals in the mouth. You cannot place copper and zinc in a battery with water, but one pole stands at a higher potential than the other element, and that produces the decomposition. There is one of the points. If you will oxidize the other point, you get no current whatever. Take dentine, for instance, and fill it with charcoal. If you impress into it those fine particles, the oxygen would have nothing to do with that tooth. It is the same thing when you take nitrate of silver. It is bringing the positive character of the dentine into a negative state. It is the potential that you did not understand. I would introduce into a canal nitrate of silver, if it were not for the color, and leave it there, not as an antiseptic, but as a permanent oxidized material that the oxygen would not touch. Acid is a portion of the chain of action of oxygen, but potential is the effect of one metal on another.

*Dr. Bogue.*—If a zinc and carbon battery be made, the zinc has a certain amount of latent power which is set in action by the presence of carbon in the fluids. I understand the potential to be the ability to enforce that power. It means the unbalancing of those two elements, and as those two metals become oxidized, the balance is restored.

*Dr. Palmer.*—Take two metals and place them in a battery, and the negative plate is of a higher potential than the positive. There they stand like a water-tank on a building, to put out fire. When you connect the wire from copper to zinc a current flows from copper to zinc pole. It will continue to act upon the zinc until it is oxidized, and the acid dissolves the zinc, and the current generated from the zinc, passing through a circuit, does the work. Take porous dentine, and put in something that will raise it to a potential as high as the filling that comes in contact with it, and then there is no trouble.

*Dr. Bogue.*—You would not place a gold filling and a tin filling in the same tooth?

*Dr. Palmer.*—In combination fillings of gold and tin there is no current passing from one to the other in a tooth.

*Dr. Bogue.*—I mean a gold filling in one surface and a tin filling in the other.

*Dr. Palmer.*—No harm if separated by enamel a line or more; if nearer than that, connect the fillings. I could go on and enumerate many influences in regard to the mouth. I could convince any of you that if you take a gold filling in one hand, and an amalgam filling in the other, I could tell you in which hand you had the gold or amalgam. That is going further than telling it in the mouth. It goes through the body; such currents do no harm. Take two adjacent teeth and get the gold and amalgam there, and make a circuit through from one to the other, and you have trouble.

*Dr. Bogue.*—What is the trouble that will occur then?

*Dr. Palmer.*—It is an irritation of the pulp that is very serious, and I have had to remove a number of them. Also from bridges jutting up against an amalgam filling. That produces an irritation of the nerves.

*A Member.*—Which tooth is irritated, the gold or the amalgam filling?

*Dr. Palmer.*—The one nearest the pulp. It will decay most around the gold filling. There is a great deal of action on an amalgam filling. Even two amalgam fillings against each other will cause a great irritation. I do not like to put two amalgam fillings close to each other, on account of the galvanic current passing from one to the other. I would rather use some other material. There is much more in this than I can explain here to-night, of the action between approximate teeth.

*Dr. Davenport.*—In an essay that Dr. Palmer read four or five years ago, entitled "Matter and Force in the Oral Cavity," I understood him to say that a gold and an amalgam filling in the same tooth, or a gold and a tin filling in the same tooth that were not connected with each other, would disintegrate the intervening tooth-substance, and he recommended connecting them. That seems to be slightly different from that he told Dr. Bogue to-night. Am I correct?

*Dr. Palmer.*—If one is on the buccal surface and the other on another surface, I would not mind it if they were sufficiently apart, so there would not be much current. It is only when they are very close together. It will dissolve the enamel between the two and run together; but when you unite the two, the amalgam filling is better than one without the gold. This sets you right in what you ask me.

A vote of thanks was offered to the essayists for their papers, and the meeting then adjourned.

John I. Hart, D.D.S.,
*Editor New York Odontological Society.*

